# Effects of Emergency Department Training on Buprenorphine Prescribing and Opioid Use Disorder-Associated ED Revisits: Retrospective Cohort Study

**DOI:** 10.5811/westjem.35589

**Published:** 2025-03-24

**Authors:** Anna Torchiano, Brian Roberts, Rachel Haroz, Christopher Milburn, Kaitlan Baston, Jessica Heil, Valerie Ganetsky, Matthew Salzman

**Affiliations:** * Cooper Medical School of Rowan University, Camden, New Jersey; † Cooper University Health Care, Department of Emergency Medicine, Camden, New Jersey; ‡ Cooper University Health Care, Cooper Center for Healing, Camden, New Jersey

## Abstract

**Introduction:**

Prescribing patients buprenorphine from the emergency department (ED) is recommended by multiple organizations. However, it is unclear how best to encourage physicians to prescribe buprenorphine from the ED. Our objectives in this study were to examine the effects of a departmental-wide training initiative for emergency physicians to prescribe buprenorphine, increase buprenorphine prescribing, and decrease ED re-utilization for opioid use disorder (OUD) complications.

**Methods:**

We performed this retrospective cohort study at an academic medical center. Beginning May 1, 2018, the ED started a buprenorphine-education initiative and tracked the proportion of clinicians who obtained buprenorphine-prescribing certification over the following 16 months. We identified adult patients referred to an addiction clinic from the ED during this period. Our primary outcome was the proportion of patients who received a buprenorphine prescription from the ED. Secondary outcomes included ED re-utilization for OUD complications and buprenorphine refills, as well as follow-up in the bridge clinic within 30 days.

**Results:**

The proportion of physicians eligible to prescribe buprenorphine increased from 37% to 88% over the study period, and 430 patients were referred to an addiction clinic. The proportion of patients referred to a bridge program who received a buprenorphine prescription increased from 50% during the first month compared to 92% during month 16 (odds ratio 1.14, 95% confidence interval 1.08–1.21 per month). There were no statistically significant changes in any secondary outcomes.

**Conclusion:**

Our intervention increased buprenorphine prescribing by emergency physicians. It did not decrease ED reutilization for complications related to opioid use disorder.

## INTRODUCTION

Opioid use disorder (OUD) and associated complications continue to be a major reason for emergency department (ED) visits across the United States. Mortality rates after ED visits for non-fatal opioid overdose are high, with an estimated 1% of patients dying in one month and 5% of patients dying within a year after discharge from the ED.^1^ Use of the ED by patients with OUD has been consistently increasing, with opioid-related ED visits doubling over the past 10 years.^2^,^3^ These trends are mainly due to patients with OUD relying heavily on the ED for most of their healthcare needs. This group of patients tends to be marginalized, with increased rates of homelessness and low socioeconomic status. These are important contributors to decreased access to primary care services and delayed treatment.^4^ Therefore, addressing OUD during ED encounters has been recognized as a unique and critical opportunity to initiate medication for opioid use disorder (MOUD) and link patients to ongoing care.

Historically, emergency physicians (EP) have not provided prescriptions to patients interested in MOUD, instead serving as a linkage to outpatient addiction services, such as a primary care physician who is able to prescribe MOUD or refer to an addiction specialist.^5^ However, a seminal study published in 2015 demonstrated that ED buprenorphine prescribing with linkage to ongoing care was associated with 78% of patients retained in outpatient addiction treatment, compared to 37% of individuals receiving referral alone and 45% receiving a brief intervention in the ED at 30 days from the ED encounter. Buprenorphine initiation in the ED also significantly decreased the use of inpatient addiction treatment services.^6^ Subsequent research has shown similar results in regard to long-term MOUD success.^7^ Many professional organizations, including the American College of Medical Toxicology and the American College of Emergency Physicians now strongly endorse this practice in an effort to expand access to addiction treatment services.^5^,^8^

Despite the evidence supporting prescribing buprenorphine within the ED, the practice has not been adopted universally across the country.^9^ Emergency physicians are often uncomfortable prescribing buprenorphine due to lack of experience, and clinicians interested in prescribing buprenorphine must go through eight hours of additional training and register with the US Drug Enforcement Administration (DEA). These barriers, along with the lack of outpatient follow-up, are common reasons for the hesitancy to incorporate buprenorphine into standard practice.^9^–^12^ To combat the lack of outpatient follow-up, many EDs and outpatient addiction medicine clinics are working together to form bridge programs, in which patients seen in the ED are provided with a referral and scheduled appointment to the addiction medicine clinic, usually within days from ED presentation.^13^–^15^

Buprenorphine prescriptions initiated in the ED have only recently gained attention. Few studies have been published regarding initiatives to increase physician buprenorphine prescribing. Our objectives in this study were to examine the effects of a departmental-wide initiative to receive certification to prescribe buprenorphine and ED re-utilization for OUD complications.

## METHODS

### Study Design and Setting

We performed a retrospective cohort study at an academic medical center, Cooper University Health Care, in Camden, NJ. Our institution developed a bridge program for patients with OUD in 2018. The bridge program is a referral system in which patients with OUD are referred by an EP to an outpatient addiction medicine clinic. Patients are referred to one of five addiction medicine clinics within Camden County, two of which are part of the study institution. All patients referred to an addiction clinic from the ED have an appointment scheduled prior to ED discharge. Available clinic appointments are posted in the ED. Patients are assigned a specific appointment by the treating physician without the need to contact a clinic directly. Clerical staff send the sign-up sheets to the respective clinic at the end of the day. Patients are also informed that if they miss their appointment, they can walk in during clinic hours for a new appointment. While the bridge program is available for patients with any substance use disorder, in this study we aimed to examine the impact of buprenorphine training on physicians and patients with OUD.

**Population Health Research Capsule**

What do we already know about this issue?

*Complications associated with opioid use disorder are a major reason for ED visits. Buprenorphine administration, prescribing, and linkage to care in the ED is safe and effective.*

What was the research question?

*Does departmental buprenorphine training increase prescribing and impact reutilization for opioid use disorder-related complications?*

What was the major finding of the study?

*Buprenorphine prescribing eligibility increased (37–88%) as did bridge referrals (50–92% [OR 1.14, 95% CI 1.08–1.21]).*

How does this improve population health?

*Buprenorphine access is limited. Departmental training increases physician prescribing and buprenorphine access for individuals with opioid use disorder.*

The institutional review board at our institution approved this study with waiver of informed consent. This study is reported in accordance with the Strengthening the Reporting of Observational Studies in Epidemiology (STROBE) Statement (Supplemental Material).

### Participants

The study included all adult patients referred to a bridge clinic by an EP from May 1, 2018–September 30, 2019. Inclusion criteria were as follows: 1) adult patients (≥18 years of age); 2) evaluated as a patient in the ED; and 3) EP referral to an addiction clinic for OUD during index ED visit. Patients were included regardless of etiology for index ED visit. We used ED bridge clinic referral records to identify potential subjects. If patients were referred to a bridge clinic multiple times within the study period, only the first referral was included in data collection. Follow-up data was only available for those individuals referred to a bridge clinic affiliated with the ED’s institution. Because three of the referral clinics were not directly affiliated with the ED institution, individuals referred to these clinics were not included in the analysis as these clinics use a different electronic health record (EHR) and there was no mechanism in place to follow these individuals longitudinally.

### Intervention

As part of a quality improvement initiative, our ED Division of Toxicology and Addiction Medicine provided a no-cost educational program to encourage EPs at our institution to meet DEA requirements and become comfortable appropriately prescribing buprenorphine from the ED. All physicians were required to complete this training to prescribe buprenorphine. Our initiative consisted of a no-cost hybrid educational seminar offered multiple times, aimed at obtaining the required training for buprenorphine prescribing. In-person sessions were four hours long and consisted of didactic lectures and small-group discussions. An additional four hours of educational sessions were asynchronous and online. The in-person sessions were led by the addiction medicine team, consisting of EPs and medical toxicologists, as well as a fellowship-trained addiction medicine specialist. Sessions were offered during ED faculty meetings and other various times to work around EP schedules.

The initiative was announced and encouraged by the ED chair during monthly faculty meetings and through email reminders. Additionally, an addiction medicine curriculum was created by emergency medicine (EM) residents in consultation with addiction medicine faculty, and all EM residents complete buprenorphine training prior to graduation. Prior to this initiative, our institution’s addiction medicine consult service was available for prescribing MOUD upon ED discharge.

### Data Collection

We tracked the proportion of EPs at our institution who were X-waivered over time. Patient data was collected from our EHR Epic (Epic Systems Corporation, Verona, WI). We used Epic Care Everywhere, a continuity-of-care document interchange between hospitals and organizations across the country that use the Epic EHR, which allows sharing of clinical information between EHRs to assess for ED reutilization. We have previously used this methodology to identify ED admissions to any hospital in the country that uses Epic Care Everywhere.^16^ The index visit was defined as the ED visit in which the patient was referred to the bridge clinic.

Two investigators independently reviewed the EHR for each subject and abstracted the data. Both abstractors had previous experience using Epic and underwent a formal training session, including performing joint data extraction on a set of practice medical records to ensure uniform handling of data. A standardized data extraction form and predefined definition of variables were used for all data collection. The abstractors held periodic meetings to review coding rules and to monitor performance.^17^ We calculated inter-observer agreement using the kappa statistic between the two abstractors. For any discrepancies, the chart was reviewed by both abstractors and consensus was reached. If a consensus was not clearly reached, we planned to have a third reviewer review the case; however, the process for handling disagreement was not required.

Abstracted data included demographics, comorbidities, additional substance use history, the etiology of the index visit, and administration of MOUD in the ED. We also determined the number of ED visits for each patient in the six months prior to the index ED visit, as well as the time from index ED visit until scheduled appointment.

### Outcome Measures

Our primary outcome was the proportion of patients with OUD referred to a bridge clinic who were prescribed buprenorphine at the index visit. We also recorded the daily dose (in milligrams [mg]) of buprenorphine prescribed for each patient. Secondary outcomes included the following: 1) ED reutilization (ie, repeat ED visit after the index visit) for opioid complication (ie, withdrawal or overdose) in the six months following the index visit; 2) ED reutilization for buprenorphine refill during the six months after index ED visit; and 3) successful follow-up at the bridge clinic within 30 days of the index visit. To determine ED reutilization we used Epic Care Everywhere as described above. We defined successful follow-up at a bridge clinic within 30 days, as opposed to scheduled appointments, given our clinic allows walk-ins and that many patients with OUD have barriers to making specific appointment times.

As part of standard operating procedure, our bridge clinic maintained a spreadsheet that listed referred patients, their appointment date, and treatment encounter dates, which was provided to the study team. Emergency department patients are referred to one of five local addiction clinics. Data was available for three of the local clinics. We excluded from this analysis subjects referred to the other two clinics. We entered all data into a Research Electronic Data Capture (REDCap, Vanderbilt University, TN) database,^18^ hosted at Cooper University Health Care and exported it into Stata/SE 16.1 for Mac (StataCorp, LP, College Station, TX) for analysis.

### Data Analysis

We reported continuous variables as mean and standard deviation or median and interquartile range (IQR) depending on data distribution. We reported categorical variables as frequency and percentages. We used a *t*-test or Wilcoxon rank-sum test to compare continuous variables, and the Fisher exact test to compare categorical variables between patients who did and did not receive a buprenorphine prescription from the ED.

We graphed the frequency of patients referred to an addiction clinic over each study month (every 30 days). We also graphed the proportion of physicians with X-waivers and the proportion of patients who received a buprenorphine prescription from the ED over each study month. For our primary outcome, we used logistic regression to calculate the odds ratio (OR) for receiving a buprenorphine prescription by study month. We also report the OR across the entire 16-month period (ie, OR calibrated for the 16-month change). We used linear regression to test whether prescribed buprenorphine dose (mg) and/or prescription length (days) increased over the study period.

For the secondary outcomes we used a Cox proportional hazards model to calculate adjusted hazard ratios (aHR) with time to ED reutilization for OUD complications as the dependent variable. We entered buprenorphine prescription at index ED visit (yes/no) as the independent variable of interest and adjusted the model for 1) ED visits in the six months prior to index ED visit, and 2) history of co-occurring substance use. We used logistic regression to calculate the OR for successful clinic follow-up as the dependent variable. We entered study month as the independent variable of interest and adjusted for lag time until scheduled clinic appointment (in days). We repeated the Cox proportional hazards model analysis for time to ED reutilization for buprenorphine refill.

## RESULTS

**Figure 1:**
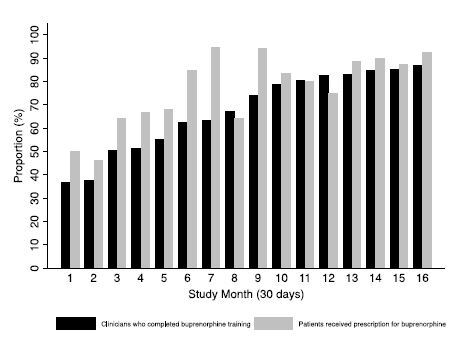
Proportion of physicians who completed training for prescribing buprenorphine and proportion of patients who received a prescription for buprenorphine per month over time.

Forty-one EPs were employed at the study institution during the study period. The proportion of EPs eligible to prescribe buprenorphine increased over the study period from 37% during the first month to 88% at the end of the study (Figure 1).

**Figure 2:**
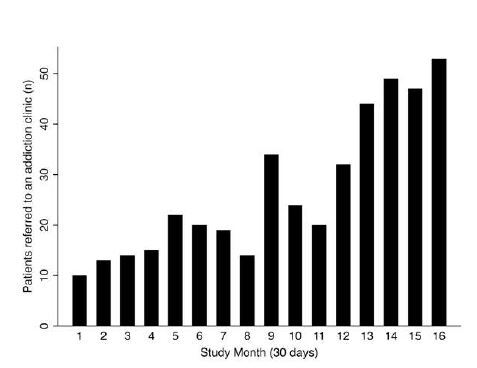
Frequency of patients referred to the bridge clinic per month over time.

A total of 430 patients were referred to an addiction clinic and were included in the study. The number of patients referred to an addiction clinic increased over time (Figure 2).Of the 430 patients, 133 were female (31%). The mean (SD) age was 38 (10) years. Most patients were White (241/430, 56%), and 115 were Black (27%). Sixty-seven patients were Hispanic (16%). Co-occurring substance use was present in 186 patients (43%) (Table 1). There was no significant difference in demographics between patients who received a buprenorphine prescription and patients who did not receive a buprenorphine prescription. Most patients (66%) had an ED visit in the six months prior to the index ED visit. The characteristics of the index visits are detailed in Table 2. Overdose was the most common cause for the index visit (37%). Less than one-third of patients were administered buprenorphine in the ED (30%), and there was no difference in the proportion of patients who were administered buprenorphine in the ED among those who received a prescription for buprenorphine compared to those who did not receive a prescription. For our primary outcome, the proportion of patients who received a buprenorphine prescription increased over the course of the study (Figure 1), from 50% in the first month to 92% in month 16 (OR 1.14, 95% confidence interval [CI] 1.08–1.21 per month). Over the entire study period the odds of receiving a buprenorphine prescription increased over 600% (OR 7.49 [95% CI 3.21–17.32]). Of the patients prescribed buprenorphine, the median (IQR) daily dose was 16 (8–16) mg and increased over time (0.30 [95% CI 0.20 to 0.40] mg per month). The median (IQR) prescription length was 6 (4–7) days and did not change over time (0.03 [95% CI −0.03 to 0.10] days per month).

Emergency department reutilization for OUD complications was recorded in 183 patients (43%). The median (IQR) time to ED reutilization for OUD complications was 41 (11–84) days. There was no change in ED reutilization for OUD complications or medication refill by study month (Supplemental Figure 1). In our multivariable model, we did not find an association between ED reutilization for OUD complications and study month when adjusting for potential confounders (aHR 0.99, 95% CI 0.96–1.02) (Supplemental Table 1). Neither did we find a difference in ED reutilization for medication refills (aHR 0.99, 95% CI 0.93–1.04) (Supplemental Table 2). Patient history of prior ED visits and co-occurring substance use was associated with ED reutilization. The kappa statistic for inter-rater agreement for ED reutilization for OUD was 0.77 (0.71–0.83).

Data for follow-up at the bridge clinic was only available for 336 patients (78%), as 94 patients were referred to bridge clinic sites that did not have appointment information available. Of those 336 patients, 151 had successful follow-up (45%). Most patients had their follow-up appointment scheduled within seven days (Table 2), and all patients had an appointment scheduled within 22 days. We did not find an increase in the proportion of patients who had successful clinic follow-up over the study period (aOR 0.95, 95% CI 0.91–1.00). Neither study month nor the number of days to the clinic appointment from the index visit were associated with successful follow-up (Supplemental Table 3).

## DISCUSSION

Our results suggest that buprenorphine training is integral to ED patients receiving buprenorphine, with an increasing proportion of physicians certified to prescribe buprenorphine being associated with increased rates of buprenorphine prescriptions. We found both the number of patients referred to an addiction clinic and the proportion of those patients who received a buprenorphine prescription increased over the study period. This is congruent with other studies assessing the barriers to ED buprenorphine utilization. Physicians are required to attend eight hours of education to prescribe buprenorphine. Our ED buprenorphine training intervention made this training more easily accessible for clinicians and increased the proportion of patients receiving a buprenorphine prescription from the ED.^19^,^20^

Our results demonstrated that ED buprenorphine prescribing was not associated with decreased ED reutilization for overdose or withdrawal, suggesting that substance use disorders are extremely complex to treat and likely involve many confounding variables, including social determinants of health. However, this study was not designed to determine such confounders. We also found that ED reutilization for buprenorphine refill did not increase over the study period, decreasing concerns that patients will use the ED for ongoing buprenorphine prescribing.

While we found that the number of patients referred to an addiction clinic increased over the study period, the proportion of patients with successful follow-up to the bridge clinic remained unchanged. It is likely that ED buprenorphine prescribing is only one part of improving OUD outcomes. Other studies have demonstrated that prescribing buprenorphine in general is associated with decreased OUD complications, such as overdose and hospitalizations.^21^–^23^ Our study failed to replicate these findings. Our differences in outcomes may be a result of social risk factors that may be different in our patient population, including transportation and housing issues, but our study was not designed to account for these risk factors. It is important to remember, however, that the ED remains the point of entry to the healthcare system for many patients with OUD. Therefore, studies examining ED utilization and buprenorphine prescribing may not be replicated nor be applicable to other settings in the healthcare system.

This is one of the few retrospective cohort studies that addresses how ED buprenorphine prescriptions affect subsequent ED usage. A similar study, conducted in 2021 by Sullivan and colleagues, showed that patients significantly reduced their ED usage after attending a bridge clinic.^24^ However, many of these patients were not referred to the clinic by EPs, and those who were referred from the ED did not have details of their intervention visit recorded. Another similar study by Le et al in 2021 also showed that ED-initiated buprenorphine was associated with lower ED utilization and hospitalization rates but did not involve a bridge program referral in the treatment course.^25^

## LIMITATIONS

Our study had several limitations. First, this study was retrospective and performed at a single site. The retrospective design did not allow identification of all patients with OUD. Further, we specifically looked at the subset of patients referred to an ED bridge program because this subset of patients were those identified by clinicians as patients seeking help for OUD. Further, all patients prescribed buprenorphine should be provided follow-up resources. Even among those seeking help and referred to an addiction clinic we found only half were prescribed buprenorphine prior to the intervention, and the number of patients referred to the clinic and the proportion of these patients who were prescribed buprenorphine increased after the intervention.

Second, we found that the number of patients referred to an addiction clinic increased over time; however, given we did not have data on the total number of patients with an OUD who presented to the ED, it is unclear whether this increase in clinic referral was due to physicians referring a higher proportion of patients with OUD to a clinic or to the absolute number of patient with OUD presenting to the ED increased. Third, we were only able to obtain information for bridge clinic follow-up for three of the five clinic sites as there was no data-sharing possible with the other two sites. Therefore, our results showing no change in the proportion of successful follow-up over the study period is limited. It is possible that patients referred to a clinic had successful follow-up at a different clinic outside our included clinics.

Fourth, increased awareness of buprenorphine prescribing outside the intervention training may have led to the increase in buprenorphine utilization in the ED. Further, new faculty joined our program who had already been trained in and had experience with prescribing buprenorphine, which may also have led to a rise in buprenorphine prescribing as opposed to the internal training. Although our results are limited to patients referred to an addiction clinic, we believe they are important and suggest that training emergency clinicians on buprenorphine use increases buprenorphine utilization. These results provide scientific rationale for future prospective studies evaluating the effects of physician training on a more generalizable population of OUD.

## CONCLUSION

Our intervention increased the number of physicians with training on buprenorphine use, which was associated with increased buprenorphine prescribing. We did not find an increase in return visits for medication refills or a decrease in ED reutilization for opioid complications. Further prospective research is needed to determine drivers of follow-up and treatment adherence, as well as the association between ED buprenorphine dosing compared to prescription alone and the association with treatment retention.

## Supplementary Information



## Figures and Tables

**Table 1:** Baseline patient characteristics.

Variables	All subjects N = 430	Buprenorphine prescription n = 354	No buprenorphine prescription n = 76	P - value
Age (years [SD])	38 (10)	39 (10)	38 (10)	0.621
Female (n [%])	133 (31)	108 (31)	25 (33)	0.683
Race (n [%])				
White	241 (56)	194 (55)	47 (62)	
Black	115 (27)	98 (28)	17 (22)	0.533
Other	74 (17)	62 (18)	12 (16)	
Hispanic ethnicity (n [%])	67 (16)	57 (16)	10 (13)	0.711
Pre-existing comorbidities (n [%])				
Diabetes	17 (4)	11 (3)	6 (8)	0.095
Hypertension	56 (13)	46 (13)	10 (13)	1
Pulmonary disease	72 (17)	58 (16)	14 (18)	0.735
Depression	67 (16)	53 (15)	14 (18)	0.486
Anxiety	72 (17)	59 (17)	13 (17)	1
Bipolar	40 (9)	31 (9)	9 (12)	0.388
Schizophrenia	8 (2)	8 (2)	0	0.361
PTSD	16 (4)	14 (4)	2 (3)	0.749
Other substances [n (%)]				
Alcohol	22 (5)	19 (5)	3 (4)	0.779
Cocaine	124 (29)	97 (27)	27 (36)	0.164
Marijuana	81 (19)	67 (19)	14 (18)	1
Phencyclidine	12 (3)	10 (3)	2 (3)	1
Any polysubstance use	186 (43)	151 (43)	35 (46)	0.611
Prior ED visit in the six months prior to index ED visit (n [%])	285 (66)	232 (66)	53 (70)	0.507
Number of ED visits in the six months prior to index ED visit* [median (IQR)]	3 (1 – 6)	3 (1 – 6)	2 (1 – 5)	0.853

* Among those with an emergency department (ED) visit in the six months prior to index ED visit (N = 285).

*IQR*, interquartile range; *PTSD*, post-trauma stress disorder.

**Table 2:** Index emergency department visit characteristics.

Variables	All subjects N = 430	Buprenorphine prescription n = 354	No buprenorphine prescription n = 76	P - value
Reason for index visit (n [%])				
Psychiatric	20 (5)	13 (4)	7 (9)	0.064
Medication refill	40 (9)	36 (10)	4 (5)	0.274
Overdose	157 (37)	132 (37)	25 (33)	0.513
Withdrawal	102 (24)	86 (24)	16 (21)	0.656
Infection	33 (8)	24 (7)	9 (12)	0.153
Other	121 (28)	97 (27)	24 (32)	0.483
Social work consult (n [%])	70 (16)	52 (15)	18 (24)	0.060
Buprenorphine administered in ED (n [%])	130 (30)	113 (32)	17 (22)	0.129
Buprenorphine total dose in ED [median [IQR])	8 (8 – 8)	8 (8 – 8)	8 (4 – 8)	0.191
Buprenorphine prescription daily dose [median [IQR])		16 (8 – 16)	N/A	N/A
Buprenorphine prescription length [days, median [IQR])		6 (4 – 7)	N/A	N/A
Naloxone prescription [IQR])	67 (16)	62 (18)	5 (7)	0.015
Days until scheduled addiction clinic appointment [median [IQR])	6 (4 – 7)	6 (4 – 7)	5 (4 – 6)	0.039
