# Supplementary material for: Effects of Emergency Department Training on Buprenorphine Prescribing and Opioid Use Disorder-Associated ED Revisits: Retrospective Cohort Study
Source: West J Emerg Med. 2025 Mar 24;26(3):580–7. doi: 10.5811/westjem.35589 (PMC12208081; doi:10.5811/westjem.35589)
Supplement: Supplementary file 1 [file wjem-26-580-s001.docx]

Supplemental materials

STROBE Checklist for cohort studies

|  | | Item No | Recommendation | Page No |
| --- | --- | --- | --- | --- |
| **Title and abstract** | | 1 | (*a*) Indicate the study’s design with a commonly used term in the title or the abstract. |  |
|  |  |  | (*b*) Provide in the abstract an informative and balanced summary of what was done and what was found. | 1-2 |
| Introduction | | | | |
| Background/rationale | | 2 | Explain the scientific background and rationale for the investigation being reported. | 4 |
| Objectives | | 3 | State specific objectives, including any prespecified hypotheses. | 5 |
| Methods | | | | |
| Study design | | 4 | Present key elements of study design early in the paper. | 5-6 |
| Setting | | 5 | Describe the setting, locations, and relevant dates, including periods of recruitment, exposure, follow-up, and data collection. | 6 |
| Participants | | 6 | (*a*) Give the eligibility criteria, and the sources and methods of selection of participants. Describe methods of follow-up. | 6 |
|  |  |  | (*b*) For matched studies, give matching criteria and number of exposed and unexposed. |  |
| Variables | | 7 | Clearly define all outcomes, exposures, predictors, potential confounders, and effect modifiers. Give diagnostic criteria, if applicable. | 6-7 |
| Data sources/ measurement | | 8* | For each variable of interest, give sources of data and details of methods of assessment (measurement). Describe comparability of assessment methods if there is more than one group. | 7-8 |
| Bias | | 9 | Describe any efforts to address potential sources of bias. |  |
| Study size | | 10 | Explain how the study size was arrived at. | 9 |
| Quantitative variables | | 11 | Explain how quantitative variables were handled in the analyses. If applicable, describe which groupings were chosen and why. | 9-10 |
| Statistical methods | | 12 | (*a*) Describe all statistical methods, including those used to control for confounding. |  |
|  |  |  | (*b*) Describe any methods used to examine subgroups and interactions. | 9-10 |
|  |  |  | (*c*) Explain how missing data were addressed. |  |
|  |  |  | (*d*) If applicable, explain how loss to follow-up was addressed |  |
|  |  |  | (*e*) Describe any sensitivity analyses |  |
| Results | | | |  |
| Participants | | 13* | (a) Report numbers of individuals at each stage of study (eg, numbers potentially eligible, examined for eligibility, confirmed eligible, included in the study, completing follow-up, and analyzed). | 10-14 |
|  |  |  | (b) Give reasons for non-participation at each stage. |  |
|  |  |  | (c) Consider use of a flow diagram. |  |
| Descriptive data | | 14* | (a) Give characteristics of study participants (eg, demographic, clinical, social) and information on exposures and potential confounders. | 12-14 |
|  |  |  | (b) Indicate number of participants with missing data for each variable of interest |  |
|  |  |  | (c) Summarize follow-up time (eg, average and total amount). |  |
| Outcome data | | 15* | Report numbers of outcome events or summary measures over time. | 12-14 |
| Main results | 16 | (*a*) Give unadjusted estimates and, if applicable, confounder-adjusted estimates and their precision (eg, 95% confidence interval). Make clear which confounders were adjusted for and why they were included. | | 12-14 |
|  |  | (*b*) Report category boundaries when continuous variables were categorized. | |  |
|  |  | (*c*) If relevant, consider translating estimates of relative risk into absolute risk for a meaningful time period. | |  |
| Other analyses | 17 | Report other analyses done—eg, analyses of subgroups and interactions, and sensitivity analyses. | | n/a |
| Discussion | | | | |
| Key results | 18 | Summarize key results with reference to study objectives. | | 4-5 |
| Limitations | 19 | Discuss limitations of the study, considering sources of potential bias or imprecision. Discuss both direction and magnitude of any potential bias. | | 14-15 |
| Interpretation | 20 | Give a cautious overall interpretation of results considering objectives, limitations, multiplicity of analyses, results from similar studies, and other relevant evidence. | | 15 |
| Generalizability | 21 | Discuss the generalizability (external validity) of the study results. | | 16 |
| Other information | | | | |
| Funding | 22 | Give the source of funding and the role of the funders for the present study and, if applicable, for the original study on which the present article is based. | | n/a |


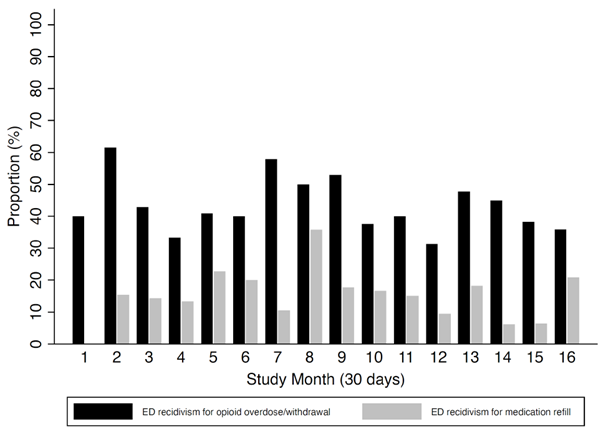


**Supplemental Figure 1.** Proportion of patients returning to the emergency department for opioid overdose/ withdrawal and proportion of patients returning for a refill of medication for opioid use disorder per month over time.

*ED*, emergency department.

**Supplemental Table 1.** Hazards ratios for emergency department reutilization for opioid overdose and withdrawal
(N = 430).

| Variables | Hazards ratio | 95% CI | *P*-value |
| --- | --- | --- | --- |
|  |  |  |  |
| Study month* | 0.99 | 0.96 - 1.02 | 0.526 |
| Previous visit | 1.44 | 1.03 - 2.02 | 0.032 |
| Co-occurring substance use | 1.53 | 1.13 - 2.06 | 0.005 |
|  |  |  |  |

*Defined as every 30 days.

*CI,* confidence interval.

**Supplemental Table 2**: Hazards ratios for emergency department reutilization for buprenorphine refill (N = 430).

| Variables | Hazards ratio | 95% CI | *P*-value |
| --- | --- | --- | --- |
|  |  |  |  |
| Study month* | 0.99 | 0.93 – 1.04 | 0.613 |
| Previous visit | 2.95 | 1.49 – 5.87 | 0.002 |
| Co-occurring substance use | 0.85 | 0.51 – 1.41 | 0.536 |
|  |  |  |  |

*Defined as every thirty days.

*CI,* confidence interval.

**Supplemental Table 3.** Odds ratios for successful bridge clinic follow-up (N = 336).

| Variables | Odds ratio | 95% CI | *P*-value |
| --- | --- | --- | --- |
|  |  |  |  |
| Study month* | 0.95 | 0.91 - 1.00 | ￼ 0.037 |
| Lag time to clinic appointment (days) | 1.02 | 0.93 - 1.11 | 0.729 |
|  |  |  |  |

*Defined as every 30 days.

*CI,* confidence interval.
